# Characterise the Response of *Amorphophallus konjac* to Different Shading Gradients Through Non-Target Metabolomics

**DOI:** 10.3390/metabo16100713

**Published:** 2026-09-24

**Authors:** Hongkun Li, Shouling Li, Siwei Pu, Xun Shi, Chunlan Meng, Shinan Gao, Shanshan Yin, Minghua Luo

**Affiliations:** Rubber Research Center, Yunnan Dehong Institute of Tropical Agricultural Science, Ruili 678600, China; lhk288@139.com (H.L.); lishouling2008@yeah.net (S.L.); 13647431003@126.com (S.P.); 18287493112@163.com (X.S.); 15636102598@163.com (C.M.); gao_sn@yeah.net (S.G.); yinshanshan92@126.com (S.Y.)

**Keywords:** *Amorphophallus konjac*, shading, metabolomics, tissue specificity

## Abstract

**Background:** Light availability governs plant growth and metabolism; however, the influence of shade intensity on metabolic allocation between leaves and corms in konjac (*Amorphophallus konjac*) remains unclear. **Methods:** Untargeted liquid chromatography-tandem mass spectrometry (LC–MS/MS) metabolomics was employed to profile metabolic responses in both tissues under three shading regimes (0%, 50%, and 75%). **Results:** A total of 1684 annotated metabolites were identified. Shading induced more pronounced changes in leaves (624 differentially accumulated metabolites) than in corms (227). These metabolites participated in oxylipin/jasmonate, lipid, flavonoid, amino acid, and organic acid metabolism. Linoleic acid metabolism was enriched in both tissues, whereas carbon/organic acid metabolism was more prominent in corms, and amino acid/flavonoid metabolism in leaves. Metabolites associated with oxylipins/jasmonates and lysophospholipids peaked at 50% shading, while flavonoids were more abundant under full light (0% shading). Among the three levels, 50% shading elicited the most significant metabolic response. Venn analysis identified 526 leaf-specific, 129 corm-specific, and 98 shared metabolites. **Conclusions:** This study establishes a metabolic framework for understanding shade responses in konjac and identifies candidate metabolites and pathways for future research on light-regulated growth and development.

## 1. Introduction

Konjac (*Amorphophallus konjac* K. Koch) is a perennial shade-tolerant herb of the Araceae family, naturally distributed in the subtropical forest understories of East and Southeast Asia [1]. It is widely cultivated as an economically important corm crop in subtropical and tropical regions owing to its exceptional richness in glucomannan (KGM), a high-molecular-weight polysaccharide. Because of its high viscoelasticity and positive health benefits, KGM has a wide range of applications in the food, pharmaceutical, and chemical industries [2,3,4]. As a typical semi-shade-tolerant plant, konjac has evolved a set of specialized physiological traits to adapt to low-light conditions, including a low light compensation point, a high specific leaf area, and an efficient light energy utilization capacity [5]. However, konjac is highly sensitive to environmental conditions, particularly fluctuations in light intensity, which poses significant challenges to large-scale cultivation. Numerous field observations and experimental studies have demonstrated that moderate shading optimizes konjac growth by alleviating photoinhibition, improving photosynthetic efficiency and biomass accumulation, and ultimately promoting corm development, thereby enhancing yield and glucomannan accumulation. In contrast, insufficient or excessive shading leads to morphological abnormalities and reduced yield, indicating that konjac has a narrow optimal light range for growth [5,6]. The balance between the beneficial and adverse effects of shading underscores the importance of elucidating the metabolic responses of konjac to varying light intensities. Despite extensive physiological research on shading responses, the core metabolic mechanisms regulating konjac adaptation to varying shading conditions remain poorly understood.

Plants can respond to fluctuations in light by modulating their secondary metabolite profiles in a systematic way. This metabolic remodelling is a key mechanism by which plants adapt to environmental stress and optimise resource allocation [7,8]. Previous studies have shown that shading treatments typically inhibit flavonoid biosynthesis while regulating amino acid and carbohydrate metabolic pathways. These changes directly reflect dynamic shifts in the plant’s carbon–nitrogen balance and redox homeostasis [9]. Furthermore, different plant tissues exhibit specific metabolic characteristics and functional differentiation, and metabolic responses induced under stress conditions often display significant tissue specificity [10]. For instance, Bénard et al.’s study on tomatoes revealed that carbon-limited plants under shading conditions could adapt to low-light environments by adjusting leaf area and reducing the carbon demand of storage organs. Notably, there were significant differences in metabolic response patterns between the source leaves and storage fruits of the plant [11]. Currently, most studies on shade-induced responses in konjac focus on physiological indicators, such as photosynthetic efficiency, chlorophyll content, antioxidant enzyme activity, and osmotic regulators [12,13]. However, traditional physiological methods monitor only a subset of functional indicators and fail to capture global metabolic changes and key regulatory metabolites involved in light adaptation. This knowledge gap significantly limits our understanding of konjac’s shade tolerance mechanisms. Therefore, systematic non-targeted metabolomic analyses are crucial to comprehensively identify small-molecule metabolite changes induced by light fluctuations and elucidate the mechanisms of metabolic reprogramming in konjac.

Metabolomics, particularly untargeted approaches using high-resolution mass spectrometry, has emerged as a powerful and unbiased strategy for comprehensively characterizing the metabolic responses of plants to environmental stimuli [14,15,16]. Previous metabolomic studies in other plant species have revealed that shading induces profound metabolic reprogramming involving primary carbon and nitrogen metabolism, as well as the biosynthesis of secondary metabolites, including phenylpropanoids, flavonoids, and terpenoids [9,17,18]. For example, Zhang et al. showed that shading significantly alters the polyphenol-to-amino acid ratio in tea plants and induces tissue-specific metabolic responses in leaves and stems [18]. Another study by Johansson et al. showed that light intensity drives gradient-dependent accumulation of sugars, tricarboxylic acid cycle intermediates, and amino acids in *Arabidopsis thaliana* [19]. Shading stress has also been shown to significantly reshape the phenylpropanoid pathway in soybean, particularly affecting isoflavone, anthocyanin, and lignin biosynthesis [9]. In saffron, exposure to low light (50% light intensity) during the seedling stage promoted carbohydrate transport from the mother corm to the leaves, reducing corm yield. During the enlargement stage of daughter corms, however, low light increased GA1 and IAA levels, decreased ABA concentration, and promoted leaf growth, thereby inhibiting carbohydrate metabolism within the daughter corms and reducing sucrose transport from leaves to daughter corms. Consequently, corm yield formation was suppressed. These findings suggest that low light alters carbohydrate distribution in saffron, disrupting the source–sink relationship and hindering the allocation of resources required for normal corm development [20]. A similar source–sink framework has been demonstrated in *Bletilla striata*, where middle light promoted polysaccharide accumulation in pseudobulbs by enhancing starch and sucrose metabolism. Sucrose produced in leaves was efficiently transported to pseudobulbs and hydrolyzed by SUS and CSLA into key precursors for polysaccharide biosynthesis, with UGP2 expression highest under middle light. In contrast, low light limited sucrose transport, while high light hindered carbohydrate degradation. These findings indicate that light intensity regulates polysaccharide accumulation in storage organs through source–sink carbon allocation and tissue-specific metabolic reprogramming, providing a basis for comparing metabolic profiles between leaves and corms in konjac [21]. However, the comprehensive metabolic consequences of differential shading on konjac, particularly the contrasting responses of photosynthetic source tissues (leaves) and non-photosynthetic sink tissues (corms), have not been systematically investigated.

In this study, untargeted liquid chromatography–tandem mass spectrometry (LC–MS/MS) metabolomics was employed to profile the metabolites of konjac leaves and corms under three shading gradients (0%, 50%, and 75% light attenuation), with the aim of investigating how shading intensity influences metabolic allocation between these two tissues. We characterized the global metabolic landscape, identified tissue-specific differentially accumulated metabolites, dissected shared and unique metabolic pathways, and screened candidate discriminative metabolites associated with tissue-specific responses. Shading induced stronger metabolic changes in leaves than in corms, with 50% shading eliciting the most pronounced response. Linoleic acid metabolism was enriched in both tissues, whereas carbon/organic acid metabolism was more prominent in corms and amino acid/flavonoid metabolism in leaves. This analysis provides a metabolic framework for understanding shade responses in konjac and identifies candidate metabolites and pathways for future research on light-regulated growth and development.

## 2. Materials and Methods

### 2.1. Plant Materials and Experimental Design

The field experiment was conducted at the experimental station of the Yunnan Dehong Tropical Agricultural Science Research Institute (97.87° E, 24.05° N; 827 m a.s.l.) during the 2025 growing season. The site has a humid subtropical monsoon climate, with a mean annual temperature of 20.7 °C, annual precipitation of 1450.2 mm, and annual sunshine duration of 2318.7 h. Second-generation corms of *Amorphophallus bulbifer* with uniform size and a fresh weight of approximately 300 g each, free from visible disease and mechanical damage, were selected as planting material. The corms were planted on 20 March 2025, in a single-ridge, double-row pattern with a spacing of 0.4 m × 0.6 m. Each plot measured 240 m^2^ and contained 1000 plants. The experiment was arranged in a randomized complete block design with three shading treatments (0%, 50%, and 75% shading), each replicated three times, giving a total of nine plots.

Basal fertilizer was applied at rates of 375 kg ha^−1^ of controlled-release balanced compound fertilizer (N:P_2_O_5_:K_2_O = 18:18:18), 300 kg ha^−1^ of calcium-magnesium phosphate fertilizer, and 30,000 kg ha^−1^ of farmyard manure. Throughout the growing period, water and fertilizer were managed via a drip irrigation system under plastic mulch. Topdressing was applied twice: in May, water-soluble compound fertilizer (N:P_2_O_5_:K_2_O = 30:10:10) was applied at 600 kg ha^−1^; in August, water-soluble compound fertilizer (N:P_2_O_5_:K_2_O = 13:7:40) was applied at 375 kg ha^−1^. Manual weeding was carried out as needed throughout the growing season. On 26 July 2025, three representative plants per plot (nine plants per treatment) with uniform growth and no pests or diseases were selected for tissue sampling. Leaf and corm samples were individually collected from each selected plant. All harvested tissues were immediately flash-frozen in liquid nitrogen and subsequently stored at −80 °C until metabolite extraction. The remaining corms were not harvested until 20 November 2025, which corresponded to the commercial maturity stage of konjac.

### 2.2. Extraction of Metabolites from Konjac

Plant samples (25 mg ± 1 mg) were weighed in a microcentrifuge tube, homogenized beads were added, and 1000 μL of extract (methanol:acetonitrile:water = 2:2:1 (*v*/*v*/*v*)) containing L-2-chlorophenylalanine-^13^C_6_ (0.05 µg/mL) as internal standard were added. The mixed solution was vortexed for 30 s, after which the samples were homogenised using a Tissuelyser–24 (Shanghai Jingxin Industrial Development Co., Ltd., Shanghai, China) at 35 Hz for 4 minutes and sonicated for 5 minutes in a 4 °C water bath. The homogenization and sonication procedure was repeated three times. The samples were incubated for 1 h at −40 °C to precipitate the proteins. They were then centrifuged at 12,000 rpm (RCF = 13,800× *g*, R = 8.6 cm) for 15 min at 4 °C. After centrifugation, 400 μL of the supernatant were transferred into the wells of a protein precipitation plate with 0.22 µm PVDF membrane (MilliporeSigma, Burlington, MA, USA). The plate was then mounted on the manifold, and a positive pressure of 6 psi was applied for 120 s. The plate was removed from the positive pressure device, and the filtrate was collected for LC-MS analysis. The quality control (QC) sample was prepared by pooling equal volumes (10 µL) of supernatant from all individual samples.

### 2.3. LC–MS/MS Analysis

LC–MS/MS analyses were performed for non-polar metabolites using a Vanquish UHPLC system (Thermo Fisher Scientific Inc., Waltham, MA, USA) coupled to an Orbitrap Exploris 120 mass spectrometer (Orbitrap MS; Thermo Fisher Scientific Inc., Waltham, MA, USA) with a Phenomenex Kinetex C18 column (2.1 mm × 100 mm, 2.6 μm, Phenomenex Inc., Torrance, CA, USA).The mobile phases were A: 0.01% acetic acid in water and B: IPA: ACN (1:1, *v*/*v*). The column temperature was 25 °C. The autosampler temperature was 4 °C, and the injection volume was 2 μL.

The Orbitrap Exploris 120 mass spectrometer was used for its ability to acquire MS/MS spectra in information-dependent acquisition (IDA) mode via the Xcalibur 4.3 acquisition software (Thermo Fisher Scientific). In this mode, the acquisition software continuously evaluates the full scan MS spectrum. The ESI source conditions were set as follows: sheath gas flow rate: 50 Arb; aux gas flow rate: 15 Arb; capillary temperature: 320 °C; sweep gas: 1 Arb; vaporizer temperature: 350 °C; full MS resolution: 60,000; MS/MS resolution: 15,000; collision energy: SNCE 20/30/40; spray voltage: 3.8 kV (positive) or −3.4 kV (negative).

### 2.4. Quality Control, Data Preprocessing, and Annotation

A total of 18 biological samples were analyzed, with seven QC samples interspersed across the analytical sequence: three at the start for system conditioning, one after every six biological samples (three injections in total), and one at the end to monitor signal drift. Blank samples consisting of pure extraction solvent were injected at regular intervals to assess potential carryover. Instrument stability was monitored by examining variations in internal standard peak height within QC samples. Analytical reproducibility was assessed using four complementary metrics: principal component analysis (PCA) to evaluate QC clustering, QC sample scores falling within ±2 standard deviations of the PCA model, pairwise correlation analysis among QC samples (R^2^ > 0.85), and calculation of the relative standard deviation (RSD) for internal standard responses, where a median RSD ≤ 15% was defined as acceptable.

For data preprocessing, individual features were first filtered to remove noise, and those with a relative standard deviation (RSD) exceeding 30% were excluded. Features with missing values in more than 50% of samples within any single group, or across all groups, were also removed. The remaining missing values were imputed using the half-minimum method. Subsequently, internal standard normalization was applied to correct variations arising from sample preparation and instrumental analysis [22]. The raw data were converted to the mzXML format using ProteoWizard and processed with an in-house program developed using R and based on XCMS for feature detection, extraction, alignment, and integration. The R package and BiotreeDB (V3.0) were used for metabolite identification according to previous reports [23].

### 2.5. Data Analysis

In this study, 51,230 features were detected and 1684 metabolites (here, the values from the raw data preprocessing) were left following relative standard deviation denoising. Then, missing values were replaced with half the minimum value. The final dataset, which contained information on the number of features, sample name, and normalised feature area, was imported into the SIMCA-P+ 18.0.1 software package (Sartorius Stedim Data Analytics AB, Umeå, Sweden) for multivariate analysis. The data were scaled and logarithmically transformed to minimise the impact of noise and the high variance of the variables. After these transformations, PCA (principal component analysis), an unsupervised analysis that reduces the dimensionality of the data, was carried out to visualise the distribution and grouping of the samples. The 95% confidence interval in the PCA score plot was used as the threshold to identify potential outliers in the dataset.

To visualise group separation and identify significantly changed metabolites, supervised orthogonal projections to latent structures-discriminant analysis (OPLS-DA) was applied. Then, 7-fold cross-validation was performed to calculate the values of R^2^ and Q^2^. R^2^ indicates how well a variable’s variation is explained, and Q^2^ indicates how well a variable can be predicted. To check the robustness and predictive ability of the OPLS-DA model, permutations were conducted 200 times. The R^2^ and Q^2^ intercept values were then obtained. Here, the intercept value of Q^2^ represents the robustness of the model, the risk of overfitting and the reliability of the model, and it is preferable for all of these to be as small as possible.

Furthermore, the variable importance in projection (VIP) value of the first principal component in OPLS-DA analysis was obtained. This summarises the contribution of each variable to the model. Student’s *t*-test was performed, and the resulting *p*-values were adjusted using the Benjamini-Hochberg method to control the false discovery rate (FDR). Metabolites with VIP > 1 and FDR < 0.05 were regarded as differentially accumulated metabolites (DAMs). In addition, pathway enrichment analysis was performed using commercial databases, including KEGG (http://www.genome.jp/kegg/, accessed on 10 September 2026) and MetaboAnalyst (http://www.metaboanalyst.ca/, accessed on 10 September 2026).

## 3. Results

### 3.1. Metabolic Composition of Konjac Corms and Leaves

A total of 1684 metabolites were detected across 18 corm and leaf samples. All annotated metabolites were classified into 8 superclasses, 56 classes, and 230 subclasses according to the metabolite classification system (Figure 1a). Figure 1b shows the proportional distribution of metabolites across the superclasses. The largest proportions were accounted for by shikimates and phenylpropanoids (25.475%), followed by terpenoids (20.843%), others (19.893%), and fatty acids (15.321%). Polyketides (4.335%), alkaloids (7.898%), amino acids and peptides (3.919%), and carbohydrates (2.316%) represented relatively smaller proportions. Overall, the detected metabolites covered both primary and secondary metabolism in konjac leaves and corms.

### 3.2. Multivariate Analysis of Shade-Induced Metabolic Changes

To determine whether the three shading treatments (0%, 50%, and 75% shade) altered the global metabolite profiles of konjac leaves and corms, principal component analysis (PCA) was performed separately for the two tissues. In corms, PC1 and PC2 explained 21.1% and 14.4% of the total variance, respectively, accounting for 35.5% of the cumulative variance (Figure 2a). In leaves, PC1 and PC2 explained 30.2% and 18.3% of the total variance, respectively, accounting for 48.5% of the cumulative variance (Figure 2b). Biological replicates from the same treatment were grouped closely in both tissues, and samples from the three shading treatments occupied distinct regions of the PCA space. Notably, the separation between treatment groups was more pronounced in leaves than in corms.

### 3.3. Identification and Tissue-Specific Distribution of Differential Metabolites

To define metabolites associated with the different shading treatments, pairwise comparisons were performed within each tissue. In corms, comparison of 0% and 50% shading identified 196 differentially accumulated metabolites (DAMs), including 144 that increased and 52 that decreased under 50% shading. Comparison of 0% and 75% shading identified 132 differentially accumulated metabolites, with 65 increased and 67 decreased under 75% shading. Comparison of 50% and 75% shading identified 182 differentially accumulated metabolites, including 61 that increased and 121 that decreased under 75% shading (Figure 3a). More differentially accumulated metabolites were detected in leaves in each pairwise comparison. The comparison between 0% and 50% shading identified 508 differentially accumulated metabolites, including 255 increased and 253 decreased metabolites. The comparison between 0% and 75% shading identified 439 differentially accumulated metabolites, with 238 increased and 201 decreased metabolites. The comparison between 50% and 75% shading identified 397 differentially accumulated metabolites, including 189 increased and 208 decreased metabolites (Figure 3a).

Across all pairwise comparisons, 624 differentially accumulated metabolites were detected in leaves (Table S2) and 227 were detected in corms (Table S1). Venn analysis revealed that 526 metabolites were detected only in leaves, 129 only in corms, and 98 were shared between the two tissues (Figure 3b).

### 3.4. Hierarchical Clustering of Differential Metabolites

Hierarchical clustering was used to examine the abundance patterns of differentially accumulated metabolites across the three shading treatments. The 227 differentially accumulated metabolites identified in corms formed three major abundance patterns after Z-score normalization. Cluster 1 contained metabolites with the highest abundance under 0% shading, which gradually decreased with increasing shade intensity. This cluster mainly included flavonoids (e.g., kaempferol glycosides, rhoifolin, catechin, and epicatechin) and the alkaloid trigonelline. Cluster 2 comprised metabolites that reached maximum abundance under 50% shading, primarily consisting of α-linolenic acid/JA pathway metabolites—including 13(S)-hydroxyoctadecadienoic acid, 9-oxooctadecadienoic acid, and linolenic acid—as well as lysophospholipids (e.g., lysophosphatidylcholine (LPC) and lysophosphatidylethanolamine (LPE)). Cluster 3 contained metabolites that were continuously upregulated with increasing shade intensity and showed the highest abundance under 75% shading, mainly comprising organic acids (malic acid, lactic acid, and glyceric acid) along with a small number of ABA-related metabolites (Figure 4a).

The 624 differentially accumulated metabolites detected in leaves also displayed distinct abundance patterns across the shading treatments (Figure 4b). Cluster 1 mainly included carotenoids, xanthophylls, ABA-associated metabolites, and several flavonoid glycosides, which showed higher abundance under 0% shading. Cluster 2 primarily consisted of oxylipin-associated metabolites and membrane lipid metabolites, including JA precursors such as 8-[3-oxo-2-pentenylcyclopentenyl]octanoic acid, which peaked at 50% shading. Cluster 3 increased with increasing shade intensity and included pigment precursors and membrane lipid-related compounds.

The clustering patterns differed between tissues, but several classes of metabolites showed similar responses in leaves and corms. Flavonoid-related metabolites tended to be more abundant under 0% shading, whereas oxylipin-associated metabolites and lysophospholipids showed higher abundance under 50% shading. Organic acids showed increased abundance under 75% shading treatment in both tissues. Among the three shading levels tested, the 50% treatment produced the most pronounced changes for several metabolite groups, particularly those associated with oxylipin metabolism and membrane lipids. Biological replicates within each treatment group clustered consistently across all heatmaps.

### 3.5. KEGG Pathway Enrichment Analysis

To further elucidate the core metabolic pathways involved in the responses of konjac corms and leaves to varying shade gradients, KEGG pathway enrichment analysis was performed on the differentially accumulated metabolites identified in each tissue, and the resulting enrichment profiles were visualized as bubble plots (Figure 5). Figure 5a shows the enrichment results for corms, and Figure 5b for leaves.

In corms, the differentially accumulated metabolites were significantly enriched in pathways associated with carbon metabolism, nucleotide metabolism, linoleic acid metabolism, glyoxylate and dicarboxylate metabolism, pyrimidine metabolism, beta-alanine metabolism, and pyruvate metabolism (Figure 5a). The enrichment profile in leaves differed markedly from that in corms. In leaves, the enriched pathways were predominantly related to amino acid metabolism, secondary metabolism, and lipid metabolism, including biosynthesis of amino acids, valine/leucine/isoleucine biosynthesis, 2-oxocarboxylic acid metabolism, linoleic acid metabolism, flavone and flavonol biosynthesis, and biosynthesis of cofactors (Figure 5b).

Linoleic acid metabolism was enriched among differential metabolites from both leaves and corms. By contrast, carbon and organic acid-related pathways were prominently enriched in corms, whereas amino acid and flavonoid-related pathways were prominently enriched in leaves. These differences were consistent with the distinct metabolite abundance patterns observed between the two tissues.

### 3.6. Shared Metabolites Between Corms and Leaves

Venn analysis identified 98 differentially accumulated metabolites (Table S3) that were shared between corms and leaves under shading treatments. A heatmap was constructed to visualize the abundance patterns of these 98 shared metabolites after Z-score normalization (Figure 6). Functional classification showed that these shared metabolites included 14 oxylipin/jasmonate pathway metabolites, 14 organic acid and carbon metabolism-related compounds, 12 membrane lipids and lysophospholipids, 9 flavonoids and polyphenols, 6 hormones and terpenoids, 5 amino acid and nitrogen metabolism metabolites, and 2 nucleotides.

Among the shared metabolites, 14 were associated with the oxylipin/jasmonate pathway, representing the largest group. Under 50% shading, 13(S)-HODE was upregulated 2.42-fold in corms and 2.88-fold in leaves, whereas the jasmonic acid precursor 8-[3-oxo-2-pentenylcyclopentenyl]octanoic acid showed a 35.66-fold increase in leaves and a 3.42-fold increase in corms. Multiple linoleic/linolenic acid oxidation products, including 9-hydroxyoctadecadienoic acid and 9,12,13-trihydroxyoctadecadienoic acid, were also upregulated in both tissues. Lysophospholipids, including LPE (18:1), LPC (17:0), LPC (18:2), LPC (18:1), and NAE (18:1), were generally upregulated 2- to 3.6-fold under 50% shading in both tissues.

Flavan-3-ols such as catechin and epicatechin reached their highest levels under 0% shading, decreased to 25–31% of 0% shading values at 50% shading, and partially recovered at 75% shading while remaining lower than under 0% shading. This trend was consistent between corms and leaves. In contrast, abscisic acid (ABA) showed opposite trends between the two tissues. In corms, ABA decreased to 31% of 0% shading values at 50% shading and recovered to 61% at 75% shading; in leaves, ABA continuously declined with increasing shade intensity, reaching 21% of 0% shading values at 75% shading. A similar trend was observed for gamma-CEHC (2,7,8–trimethyl–2–(2′–carboxyethyl)–6–hydroxychromane).

Malic acid increased with greater shade intensity in both tissues, reaching 1.32-fold in corms and 1.23-fold in leaves under 75% shading. In contrast, amino acids such as glutamic acid and asparagine were significantly reduced under 50% shading in both tissues. Among the 98 shared metabolites, 66 peaked at 50% shading in corms and 45 peaked at 50% shading in leaves. Overall, several metabolic features exhibited a peak response at 50% shading, whereas other metabolites displayed tissue-dependent or monotonic changes across the shading treatments. The shared metabolite set included changes in oxylipin-associated metabolites, membrane lipids, flavonoids, organic acids, amino acids, and hormone-associated metabolites. These common changes indicate that shading affected multiple metabolic processes in both leaves and corms, while the distinct abundance patterns observed between tissues suggest tissue-specific responses.

## 4. Discussion

This study employed metabolomics to analyze the metabolic reprogramming of konjac (*Amorphophallus konjac*) corms and leaves under varying shading levels, revealing tissue-specific and multi-pathway metabolic changes, with 50% shading eliciting the most pronounced metabolic response among the three treatments. Global metabolomics analysis indicated that shikimates and phenylpropanoids (25.475%) constituted the most abundant metabolite class in the konjac metabolome, consistent with the high inherent polyphenol levels in konjac plants [24]. The predominance of phenylpropanoid metabolism agrees with previous reports, demonstrating its roles not only in konjac defense against pathogens such as *Fusarium solani* [25] but also as a core metabolic pathway in konjac [26]. Principal component analysis (PCA) showed much clearer separation among leaf samples than corm samples across shading treatments, indicating that shading triggered stronger global metabolic shifts in leaves. Quantitative results supported this pattern: 624 differentially accumulated metabolites (DAMs) were identified in leaves versus 227 in corms. This tissue-specific differentiation aligns with findings in *Amorphophallus bulbifer*, where flower and leaf buds displayed distinct metabolic profiles [27]. Comparable tissue-specific metabolic responses to shade have also been reported in tea, with differential regulation of amino acids, polyphenols, and organic acids between leaves and stems [18], as well as in soybean under shade stress [9]. Tissue-specific metabolic reprogramming appears to be a widespread phenomenon, especially between photosynthetic vegetative tissues and carbon storage sink tissues. In the medicinal fungus *Gerronema lapidescens*, mycelia and sclerotia adopt distinct metabolic strategies; sclerotia accumulate bioactive compounds and activate oxidative stress-related pathways [28]. Such divergent metabolic strategies between tissues mirror the contrasting metabolic features observed between konjac leaves and corms.

The 50% shading treatment was consistently identified as the pivotal metabolic hub based on multiple analytical strategies. Pairwise comparisons revealed that the contrast between 0% and 50% shading yielded the largest number of DAMs in both corms and leaves: 196 DAMs in corms and 508 DAMs in leaves. Of the 98 metabolites shared between the two tissues, 66 reached their maximum abundance under 50% shading in corms, and 45 peaked at this shading level in leaves. This observation corroborates prior physiological investigations reporting that 50–70% shading enhances photosynthetic efficiency and corm yield in *Amorphophallus konjac* [6], and that a 25–50% shading regime is optimal for most *Amorphophallus* species [13]. Collectively, these studies pinpoint 50% shading as a common critical threshold. Comparable findings have been documented in *Polygonatum cyrtonema*, where moderate 60% shading markedly stimulates plant growth and triggers reprogramming of carbohydrate, terpenoid, and phenylpropanoid metabolism, consequently modifying phytochemical characteristics [17].

Hierarchical clustering analysis resolved the metabolic responses into three distinct abundance patterns: metabolites that decreased with shading (full-light maximum), metabolites that peaked at 50% shading (moderate-shade peak), and metabolites that increased with shading intensity (severe-shade maximum). In this study, catechins, epicatechins, and other flavanols accumulated abundantly under full light and decreased significantly with increasing shading, with this trend consistently observed in both corms and leaves. This pattern is consistent with previous reports that enhanced photosynthetically active radiation can upregulate flavonoid synthase genes (such as CHS, F3H, and DFR), promoting flavonoid accumulation, and that flavonoids serve photoprotective functions by absorbing UV radiation and scavenging ROS [29]. Such light-mediated regulation of the phenylpropanoid–flavonoid pathway is a well-documented phenomenon across plants, as previously reported in soybean and tea [9,30].

The second pattern, the moderate-shade peak, was primarily represented by metabolites of the jasmonate/oxylipin pathway. The cyclopentane-type JA precursor, 8-[3-oxo-2-pentenylcyclopentenyl]octanoic acid, increased 35.66-fold in leaves and 3.42-fold in corms at 50% shading. The involvement of jasmonic acid (JA) in plant stress responses is well established, and increasing attention has been given to its role in shade and weak light responses. In basil, far-red light supplementation upregulates multiple hormones, including JA, gibberellin, auxin, and salicylic acid, and alters the expression of jasmonate ZIM domain-containing proteins (JAZ) in the plant hormone signal-transduction pathway, promoting shade avoidance responses [31]. In tea plants subjected to combined drought and shade treatments, JA- and abscisic acid (ABA)-signaling pathways activate aroma compound accumulation [32]. At the metabolic level, plants activate the lipoxygenase (LOX) pathway to release α-linolenic acid from membrane lipids, which is then converted into JA and its derivatives through the action of 13-LOX, AOS, and AOC. JA thus participates in both biotic and abiotic stress responses [33]. Concurrently, lysophospholipids (LPC, LPE, NAE, etc.) accumulated synchronously under 50% shading in both tissues, representing the same moderate-shade peak pattern. Lysophospholipids are products of phospholipase A-mediated hydrolysis of membrane phospholipids; they serve as markers of membrane lipid changes and participate in stress signal transduction as lipid second messengers. The plant phospholipase family plays a central role in membrane lipid metabolism, cell signal transduction, and stress responses [34]. In alfalfa, lysophospholipid accumulation has been linked to stress signal transduction, and membrane lipid remodeling is considered an important strategy for plant adaptation to abiotic stress [35].

The third pattern—metabolites that increased with shading intensity—mainly comprised organic acids, with malic acid showing progressive accumulation in both tissues (1.32–fold in corms and 1.23–fold in leaves at 75% shading). KEGG enrichment analysis further revealed that glyoxylate and dicarboxylate metabolism was significantly enriched in corms, consistent with the organic acid accumulation observed in corms under severe shading. Collectively, the tissue-specific enrichment patterns, carbon and organic acids metabolism in corms versus amino acid and secondary metabolism in leaves, are consistent with the differentiation observed in PCA and DAMs analysis, reinforcing the view that metabolic adjustments in corms are primarily centered on carbon and energy homeostasis, while leaves engage more broadly in amino acid and secondary metabolic pathways.

Several limitations should be acknowledged. The use of only three shading levels and a single time point limits our ability to define the optimal threshold or capture the temporal dynamics of metabolic reprogramming. Future studies incorporating finer shading gradients and time-series multi-omics analyses will be required to further elucidate the metabolic network associated with konjac shade acclimation.

## 5. Conclusions

In summary, this study characterized the metabolic responses of konjac leaves and corms to three shading levels using untargeted LC–MS/MS profiling. Shading altered a broad range of metabolites, including those involved in lipid and oxylipin metabolism, flavonoid metabolism, and organic and amino acid metabolism. Leaves showed a greater number of differentially accumulated metabolites and clearer separation among shading treatments than corms, highlighting tissue-specific metabolic responses to shading. Metabolite accumulation patterns fell into three distinct categories: flavonoid-related metabolites were more abundant under full light, several oxylipin-associated metabolites and lysophospholipids increased under 50% shading, and organic acids such as malic acid accumulated under severe shading. KEGG pathway enrichment further revealed that linoleic acid metabolism was enriched in both tissues, whereas carbon and organic acid metabolism were more prominent in corms, and amino acid and flavonoid metabolism contributed more strongly to the leaf response. Among the three shading levels examined, 50% shading showed the highest number of DAMs and the greatest accumulation of shared metabolites, particularly for oxylipin-associated compounds and membrane lipids. These findings provide a metabolic framework for understanding konjac shade responses and identify candidate metabolites and pathways for future mechanistic studies.

## Figures and Tables

**Figure 1:**
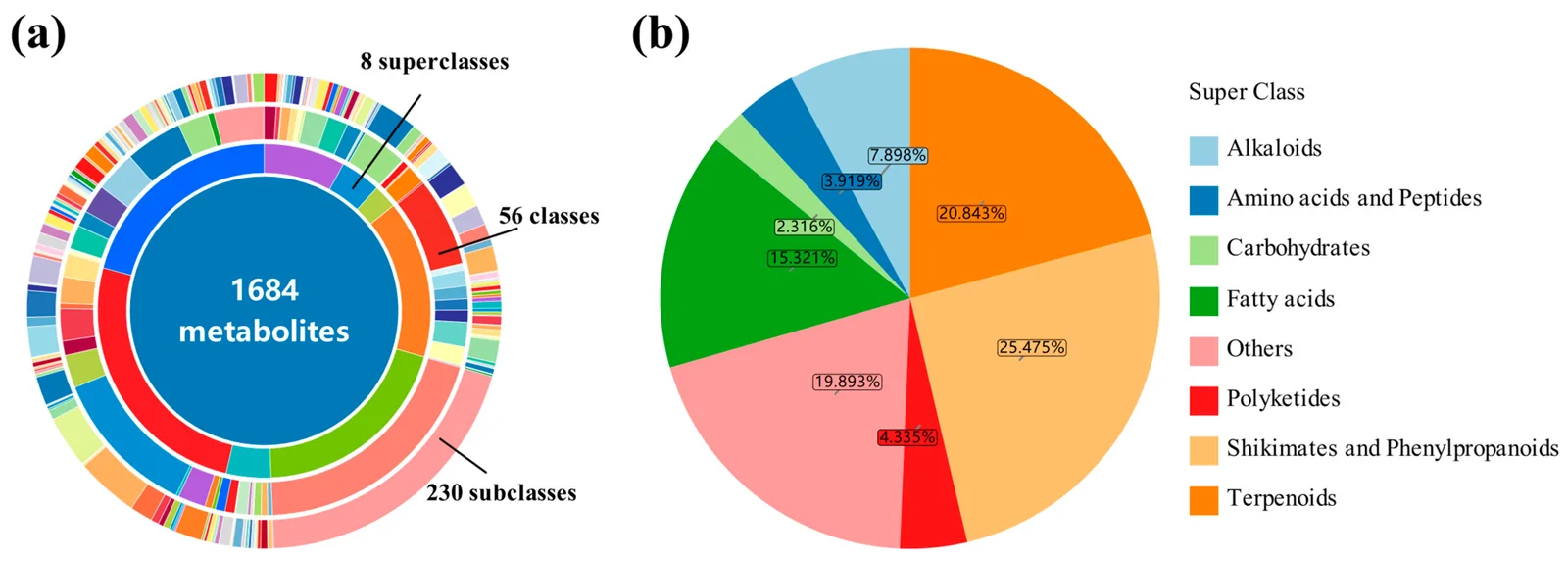
Classification statistics of annotated metabolites. (**a**) Multi-ring chart illustrating hierarchical classification of all 1684 annotated metabolites. From inner to outer circles: total metabolites, 8 superclasses, 56 classes, and 230 subclasses. (**b**) Pie chart showing the proportion of metabolites in 8 superclasses.

**Figure 2:**
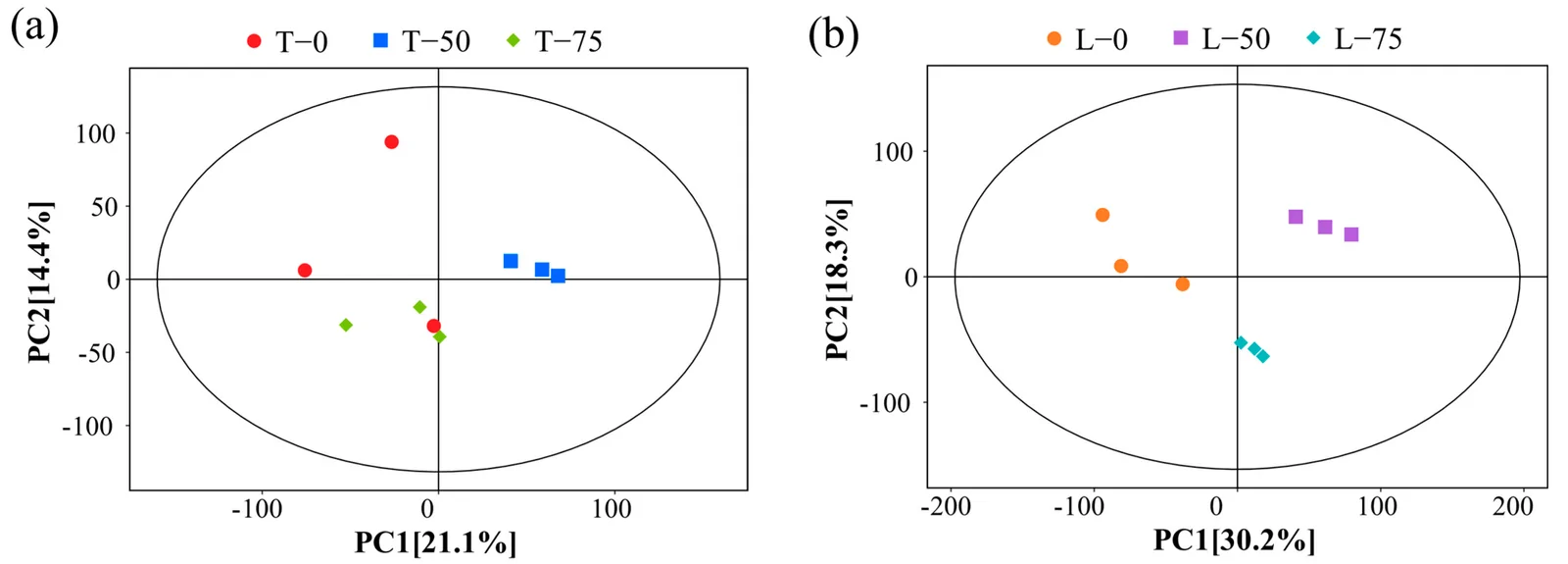
Principal component analysis (PCA) score plots of metabolome in konjac corms and leaves under different shade treatments. (**a**) PCA plot of konjac corm samples; (**b**) PCA plot of konjac leaf samples. T-0, T-50, T-75 represent konjac corms grown under 0%, 50%, and 75% shade, respectively; L-0, L-50, L-75 represent konjac leaves under 0%, 50%, and 75% shade, respectively. The percentage values on axes represent the explained variance of each principal component.

**Figure 3:**
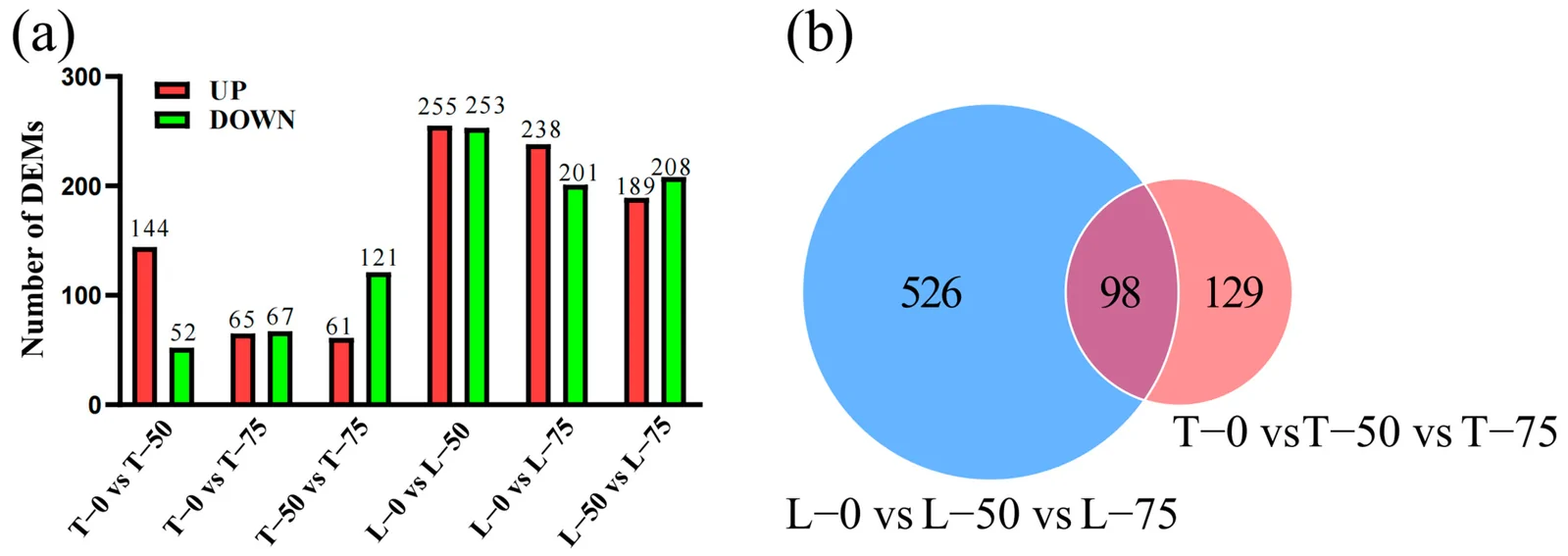
Statistics of differentially accumulated metabolites (DAMs) in konjac corms and leaves under different shade treatments. (**a**) Bar chart showing the number of DAMs in pairwise comparisons. Red bars represent significantly upregulated metabolites (UP), green bars represent significantly downregulated metabolites (DOWN). T-0/T-50/T-75 indicate corm samples under 0%, 50%, 75% shade, respectively; L-0/L-50/L-75 indicate leaf samples under 0%, 50%, 75% shade, respectively. (**b**) Venn diagram of all DAMs identified from corms and leaves. The blue region represents leaf-specific DAMs, the red region represents corm-specific DAMs, and the overlapping region corresponds to shared DAMs between corms and leaves.

**Figure 4:**
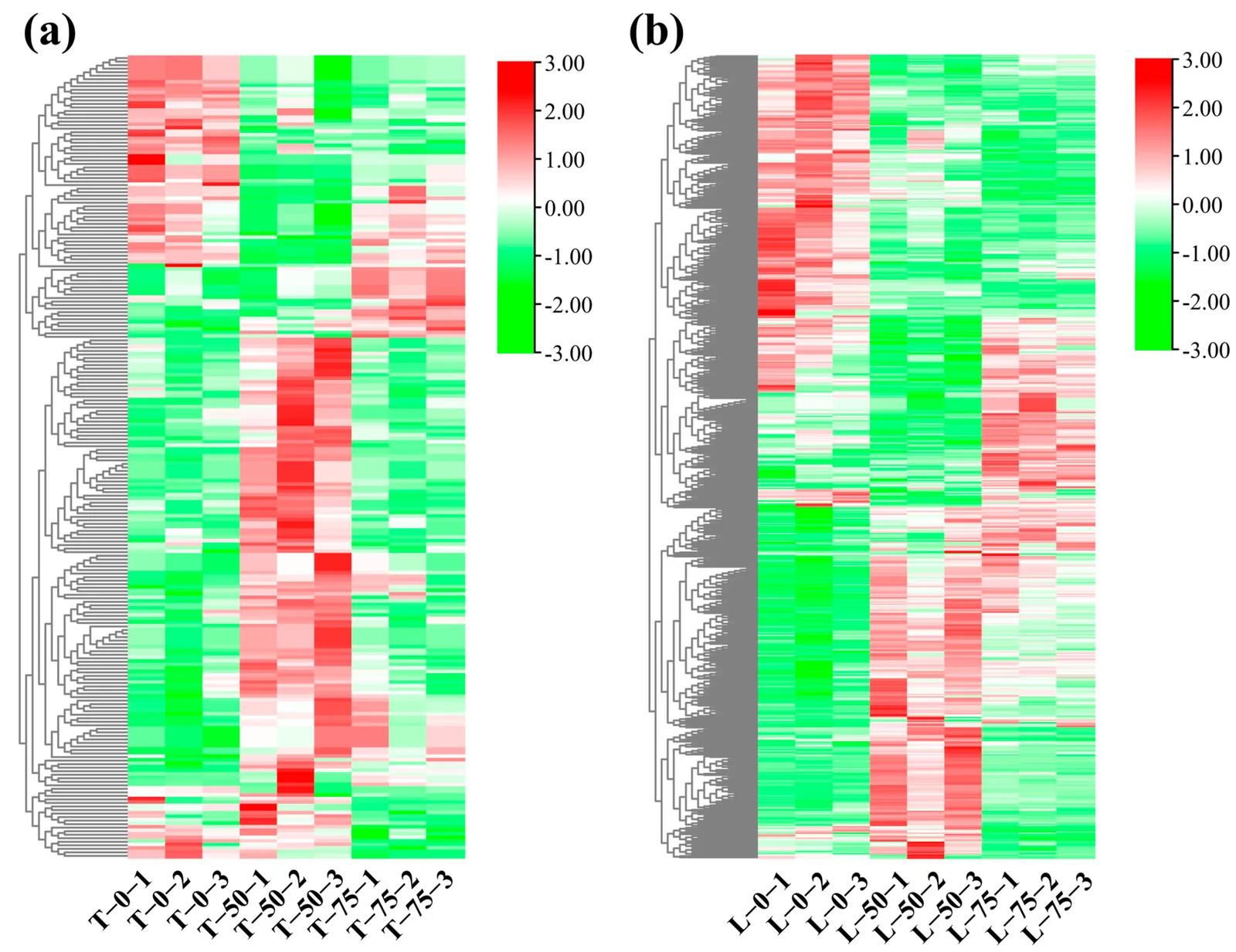
Hierarchical clustering heatmap of differentially accumulated metabolites in konjac corms and leaves under different shade treatments. (**a**) Heatmap of 227 differentially accumulated metabolites in corms; (**b**) Heatmap of 624 differentially accumulated metabolites in leaves. Rows represent metabolites, columns represent samples (T-0/T-50/T-75: corm samples under 0%, 50%, 75% shade, respectively; L-0/L-50/L-75: leaf samples under 0%, 50%, 75% shade, respectively; with 3 biological replicates per group). Color represents relative abundance after Z-score normalization; red indicates high abundance, green indicates low abundance. The dendrogram on the left shows classification of metabolite abundance patterns.

**Figure 5:**
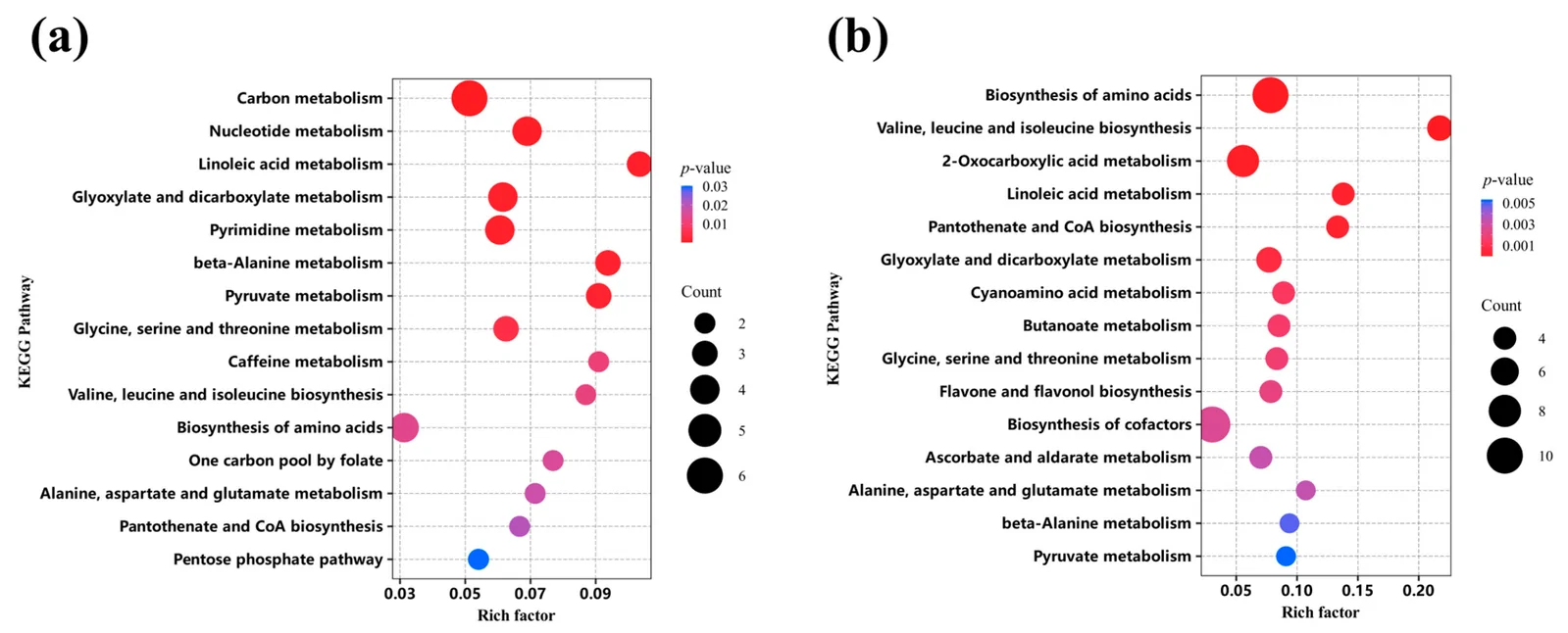
Bubble diagrams of KEGG pathway enrichment for differentially accumulated metabolites in konjac corms and leaves. (**a**) KEGG enrichment of DAMs in corms; (**b**) KEGG enrichment of DAMs in leaves. The *x*-axis represents Rich factor. Bubble size indicates the number of differentially accumulated metabolites enriched in each pathway. Bubble color corresponds to the enrichment *p*-value; redder bubbles represent lower *p*-value and higher enrichment significance.

**Figure 6:**
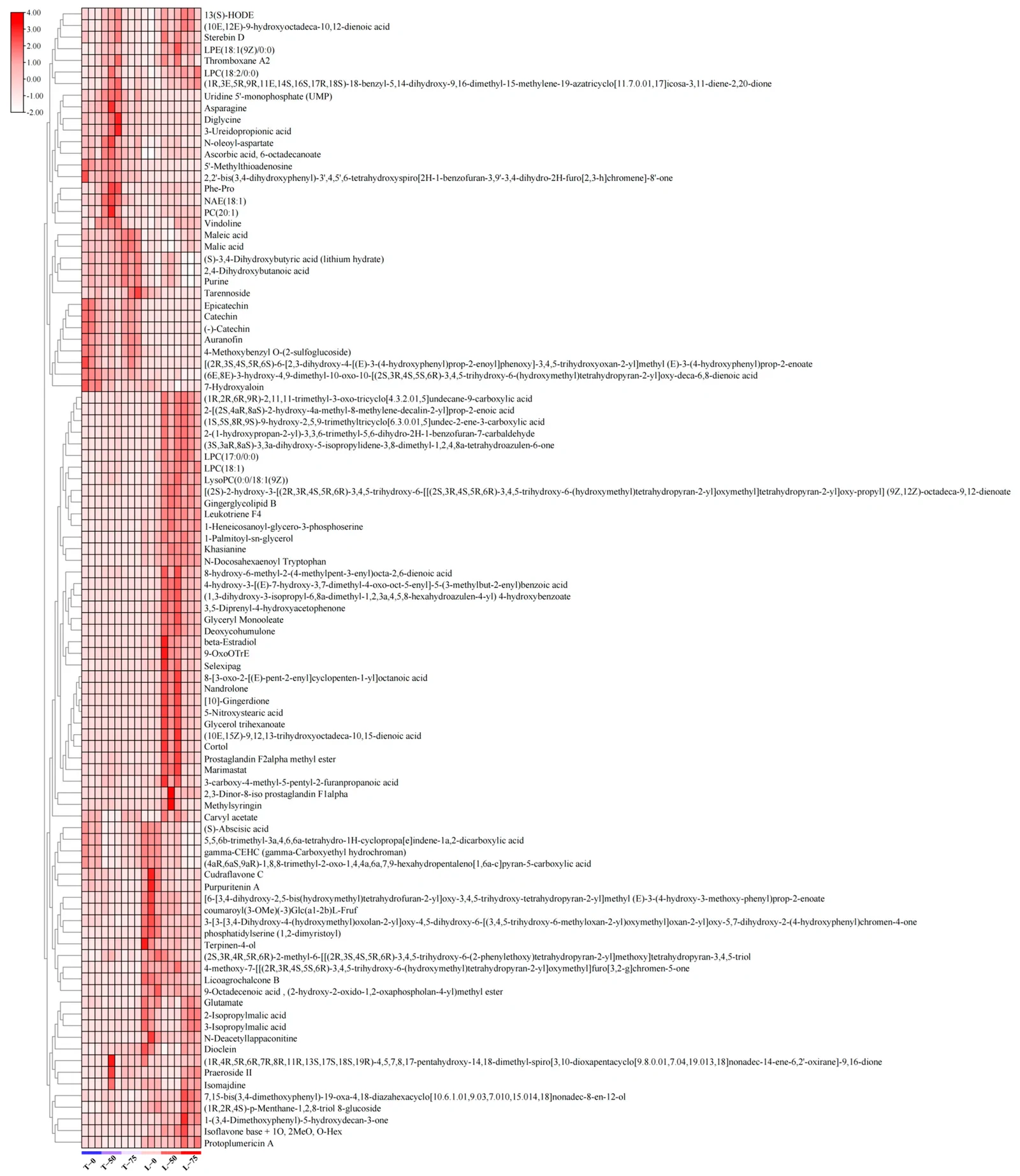
Hierarchical clustering heatmap of shared differentially accumulated metabolites in corms and leaves. Rows indicate metabolites, columns indicate samples from different tissues and shade treatments. Colors represent Z-score normalized relative metabolite abundance; red denotes high abundance and white denotes low abundance. T-0, T-50, T-75: konjac corms under 0%, 50%, 75% shade, respectively; L-0, L-50, L-75: konjac leaves under 0%, 50%, 75% shade, respectively.

## Data Availability

The study’s original contributions are outlined in the article and Supplementary Materials. If you require further information, please contact the corresponding author.

## Supplementary Materials

The following supporting information can be downloaded at https://www.mdpi.com/article/10.3390/metabo16100713/s1, Table S1: Abundance profiles of differentially accumulated metabolites in konjac corms under varying shade intensities; Table S2: Abundance profiles of differentially accumulated metabolites in konjac leaves under varying shade intensities; Table S3: Abundance profiles of shared DAMs in konjac corms and leaves.
